# Genomic characterization and phylogenetic analysis of a novel Nairobi sheep disease genogroup Orthonairovirus from ticks, Southeastern China

**DOI:** 10.3389/fmicb.2022.977405

**Published:** 2022-08-25

**Authors:** Xu Zhang, Hang-Yuan Li, Jian-Wei Shao, Ming-Chao Pei, Chong Cao, Fu-Qiang Huang, Ming-Fei Sun

**Affiliations:** ^1^School of Life Science and Engineering, Foshan University, Foshan, China; ^2^Fujian Provincial Key Laboratory for the Prevention and Control of Animal Infectious Diseases and Biotechnology, Longyan, China; ^3^Key Laboratory of Preventive Veterinary Medicine and Biotechnology, Longyan University, Longyan, China; ^4^Zhaoqing/Maoming Branch Center of Guangdong Laboratory for Lingnan Modern Agricultural Science and Technology, Zhaoqing, China; ^5^Key Laboratory of Livestock Disease Prevention of Guangdong Province, Guangzhou, China; ^6^Key Laboratory of Avian Influenza and Other Major Poultry Diseases Prevention and Control, Ministry of Agriculture and Rural Affairs, Guangzhou, China; ^7^Institute of Animal Health, Guangdong Academy of Agricultural Sciences, Guangzhou, China

**Keywords:** Meihua Mountain virus, novel Orthonairovirus, NSD genogroup, metatranscriptomics, China

## Abstract

The increasing prevalence and transmission of tick-borne diseases, especially those emerging ones, have posed a significant threat to public health. Thus, the discovery of neglected pathogenic agents carried and transmitted by ticks is urgently needed. Using unbiased high-throughput sequencing, a novel Orthonairovirus designated as Meihua Mountain virus (MHMV), was identified in bloodsucking ticks collected from cattle and wild boars in Fujian province, Southeastern China. The full-length genome was determined by RT-PCR and RACE. Genomic architecture of MHMV shares typical features with orthonairoviruses. Phylogenetic analyses suggested that MHMV is clustered into the Nairobi sheep disease (NSD) genogroup of the genus *Orthonairovirus* and is closely related to the Hazara virus. The RdRp, GPC, and N protein of MHMV shares 62.3%–83.5%, 37.1%–66.1%, and 53.4%–77.3% amino acid identity with other NSD genogroup viruses, respectively, representing a novel species. The overall pooled prevalence of MHMV in ticks was 2.53% (95% CI: 1.62%–3.73%, 22 positives of 134 tick pools), with 7.38% (95% CI: 3.84%–12.59%, 11 positives of 18 pools) in *Haemaphysalis hystricis*, 6.02% (95% CI: 1.85%–14.22%, four positives of eight pools) in *H. formosensis*, 25.03% (95% CI: 9.23%–54.59%, six positive of eight pools) in *Dermacentor taiwanensis*, and 0.16% (95% CI: 0.01%–0.72%, one positive of 100 pools) in *Rhipicephalus microplus*. This study presented the first report of tick-carried Orthonairovirus in Fujian province and highlighted the broad geographic distribution and high genetic diversity of orthonairoviruses in China.

## Introduction

Ticks are one of the main vectors transmitting viral, bacterial, and parasitic pathogens that cause severe illnesses to animals and humans. The increasing prevalence and transmission of tick-borne diseases, especially those emerging ones such as severe fever with thrombocytopenia syndrome and Heartland virus diseases, are keeping challenging our preparedness and have posed a significant threat to public health (Madison-Antenucci et al., 2020). Thus, unbiased discovery and detection of emerging human-pathogenic agents carried and transmitted by ticks are urgently needed.

Members of the genus *Orthonairovirus* are all transmitted by ticks, distinguishing themselves from most of the other members of the family *Nairoviridae* (Lasecka and Baron, 2014). Based on the phylogenetic relationships, tick-associated orthonairoviruses were classified into nine genogroups, of which the most important is the Nairobi sheep disease (NSD) genogroup (Walker et al., 2016). The NSD genogroup includes Crimean-Congo hemorrhagic fever virus (CCHFV), Nairobi sheep disease virus (NSDV), Hazara virus (HAZV), Dugbe virus (DUGV), and Kupe virus (KUPV; Walker et al., 2016). The recently identified Tofla virus (TFLV) also belongs to this genogroup (Shimada et al., 2016). Among these, the fatal human pathogen CCHFV can cause hemorrhagic fever with mortality up to 30% (Shahhosseini et al., 2021), and the NSDV causes severe hemorrhagic gastroenteritis in sheep and goats with mortality up to 90% (Krasteva et al., 2020). Both CCHFV and NSDV endemic foci can be found in Africa, Asia, and the Middle East, suggesting the wide geographic distribution of orthonairoviruses. Except for CCHFV and NSDV, the vertebrate host has not been established for other members of the NSD genogroup. Meanwhile, human infection has not been reported, thus their relevance to public health remains unclear. However, infection with these viruses can be lethal to immunocompromised mice, emphasizing their pathogenic potential (Dowall et al., 2012; Shimada et al., 2016). Importantly, several recently discovered tick-borne orthonairoviruses in Eastern Asia, such as the Yezo virus (Kodama et al., 2021), Songling virus (Ma et al., 2021), and Tacheng tick virus 1 (Liu et al., 2019), were associated with human febrile illness with different severity, suggesting the high diversity of orthonairoviruses in nature and their potential risk to public health.

With the expansion of tick habitats, it is necessary to monitor the agents they carried (Dong and Soong, 2021). Fujian, a coastal mountainous province, locates in Southeastern China and possesses a middle subtropical climate. The suitable climatic conditions and abundant wildlife in mountains shaped a perfect environment for several tick species, such as *Ixodes granulatus*, *Rhipicephalus microplus*, and *Dermacentor auratus* (Zhang et al., 2019). The Meihua Mountain Nature Reserve in Fujian province is an ecological forest area, in which a variety of wild animals provide abundant host resources for ectoparasitic ticks. However, little is known about the background information of tick-carried pathogenic agents. In this study, we performed unbiased high-throughput meta-transcriptomic sequencing to profile RNA virus in ticks. A novel member of NSD genogroup Orthonairovirus, designated as Meihua Mountain virus (MHMV), was identified in bloodsucking ticks collected from cattle and wild boar. The complete genome sequence, genomic characteristics, phylogenetic relationship, and molecular prevalence of MHMV were analyzed. This study presented the first report of tick-borne Orthonairovirus in Fujian province and highlighted the broad geographic distribution and genetic diversity of orthonairoviruses in China.

## Materials and methods

### Tick collection and RNA extraction

From 2019 to 2020, 988 bloodsucking ticks were collected from cattle and wild boar in seven villages of Meihua Mountain Nature Reserve, Fujian province, Southeastern China. The tick species were morphologically identified by microscopy, and were further confirmed by 16S ribosomal RNA gene sequencing as described (The primers used are listed in Supplementary Table S1; Sameroff et al., 2019). Subsequently, the same species of ticks were pooled and stored at −80°C until further processing.

Before extracting nucleic acid, pooled samples were washed in 1 ml of 70% ethanol followed by three washes with nuclease-free water and then dried. Each pool was homogenized in 400 μl Dulbecco’s Modified Eagle Medium (DMEM; Thermo Fisher Scientific, Waltham, MA, United States) using TissueLyser II (Qiagen, Hilden, Germany). Homogenates were then centrifuged at 12,000 rpm for 1 min to precipitate debris. To extract RNA, 100 μl supernatant was mixed with 300 μl TRIzol LS reagent (Invitrogen, Carlsbad, CA, United States) and purified according to the manufacturer’s instructions. For meta-transcriptomic sequencing, a total of 34 pools were selected based on tick species and host animals (Supplementary Table S2). The total RNA was extracted using the homogenate mixture from 34 pools according to tick numbers in the pools to make sure that the RNA from each tick is equal. All purified RNA was quantified using Qubit 3.0 fluorometer (Thermo Fisher Scientific) and stored at −80°C until further use.

### Meta-transcriptome sequencing

RNA library preparation was conducted following the library preparation protocol provided by Illumina, with slight modification. Briefly, ribosomal RNA in total RNA was removed by a Ribo-Zero Plus rRNA Depletion Kit (Illumina, San Diego, CA, United States). The remaining RNA was then fragmented, reverse-transcribed, adapted, purified, and analyzed with the Agilent 2100 Bioanalyzer. Paired-end (150 bp) sequencing was conducted on the Illumina HiSeq 2500 platform (Illumina). The library preparation and sequencing were performed at Novogene (Tianjin, China).

### Bioinformatics analysis and full-length genome determination

The sequencing reads (raw data) were quality-controlled with SOAPnuke (v2.0.5) software to generate clean reads (Chen et al., 2018). The rRNA and host reads were then filtered by BWA (v0.7.17) with default settings (Li and Durbin, 2009). *De novo* assembly was performed with Megahit (v1.1.2; Li et al., 2015b) and the resulting contigs were blasted against reference virus nucleotides and proteins by BLASTN as well as Diamond BLASTX, respectively (Buchfink et al., 2015). The viral contigs mapped to the same segment were further assembled using ContigExpress, and subsequently mapped to a Hazara virus reference genome (GenBank accession numbers are listed in Supplementary Table S3) using Geneious (Biomatters, Ltd., New Zealand).

To fill the gaps between contigs, primers were designed according to corresponding contigs. Then nested reverse transcription polymerase chain reaction (RT-PCR) was done and followed by Sanger sequencing. For the genome termini, rapid amplification of cDNA ends (RACE) was performed using a SMARTer RACE 5′/3′ Kit (Takara, Beijing, China), as described previously (Li et al., 2015a).

### Molecular screening of MHMV in individual tick pools

Primer pairs targeting the coding region of the L segment were designed. Primers are listed in Supplementary Table S1. Total RNA extracted from individual tick pools (134) was subjected to the screening of MHMV using nested RT-PCR and confirmed by sequencing. The nested PCR was performed according to the manufacturer’s instructions. Briefly, RT-PCR was performed with the PrimeScript One Step RT-PCR kit (TaKaRa). The 50 μl reaction contained 18 μl RNase-Free ddH_2_O, 25 μl 2× buffer, 2 μl PrimeScript Enzyme, 2 μl of forward and reverse primers, and 2 μg total RNA. The cycling program was set at 50°C for 30 min and 94°C for 2 min, followed by 35 cycles of standard PCR and a final extension step at 72°C for 5 min. The second round PCR was performed with ExTaq Premix (TaKaRa). The reaction was assembled by combining 19 μl RNase-Free ddH_2_O, 25 μl Taq Premix, 2 μl of forward and reverse primers each, and 2 μl PCR product from RT-PCR reaction. The following cycles were set as 98°C for 1 min, 35 cycles of standard PCR, and 72°C for 5 min. The annealing temperature of both RT-PCR and the second round PCR was set as 55°C.

Prevalence of MHMV was estimated assuming perfect sensitivity and specificity of molecular detection using the EPITOOLS online statistical program “Pooled prevalence for variable pool size and perfect tests”^1^ that based on the model established by Williams and Moffitt (2001). The input data were prepared as instructed by the EPITOOLS user guide.

### Virus isolation

The homogenate from positive tick samples was centrifuged at 6,000 *g* for 10 min and the supernatant was collected. The 100 μl of undiluted and diluted (1:5 and 1:10) supernatant was inoculated onto monolayers of Vero E6, SW-13, and BHK-21 cell in 6-well plate. After 2 h of inoculation, the supernatant was aspirated out and replaced with medium containing 2% fetal bovine serum. The cytopathic effect (CPE) was examined daily and each passage was tested for the presence of MHMV RNA using RT-PCR as described in section 2.4. Unfortunately, neither apparent CPE nor the existence of viral RNA was observed throughout eight blind passages, indicating that MHMV may not be able to propagate in the above cell lines.

### Genomic characterization and phylogenetic analysis

The potential open reading frames (ORFs) were predicted in ORFfinder and compared with other Orthonairovirus sequences (GenBank accession numbers are listed in Supplementary Table S2). Analysis of the glycoprotein precursor (GPC) domains and post-translational modification features was conducted with TMHMM (for transmembrane protein prediction; Krogh et al., 2001), ProP−1.0 (for signal peptidase cleavage site prediction; Petersen et al., 2011), NetNGlyc−1.0 (for *N*-linked glycosylation site prediction), and NetOGlyc (for mucin-type *O*-glycosylation site prediction; Steentoft et al., 2013).

To determine the phylogenetic relationship among orthonairoviruses, amino acid alignments were conducted using the E-INS-i algorithm in MAFFT 7.0 (Katoh et al., 2019). TrimAI 1.2 was used to trim off ambiguous positions and sequences were re-aligned in MEGA X (MUSCLE; Capella-Gutiérrez et al., 2009; Kumar et al., 2018). Phylogenetic trees were constructed using the maximum likelihood (ML) method (LG substitution model, Gamma-distributed rate variation) with 100 bootstrap replications (Le and Gascuel, 2008).

The amino acid and nucleotide sequence identity was determined with Clustal Omega (Sievers and Higgins, 2018). Both the putative Gn and Gc protein amino acid sequence alignment of NSD genogroup viruses were performed with MAFFT version 7.0 online server^2^ (Katoh et al., 2019). G-INS-i algorithm was used and the Unaligned level value was set as 0.4 with other parameters that were set as default. Visualization of the resulting alignments was done with the ESpriPt 3.0^3^ (Robert and Gouet, 2014) and edited in Adobe Illustrator CC.

### Ethical approval

This study did not involve the purposeful killing of animals. All samples were collected by passive surveillance under Chinese legislation. No ethical approval was necessary.

## Results

### Identification of MHMV in ticks

**Figure 1:**
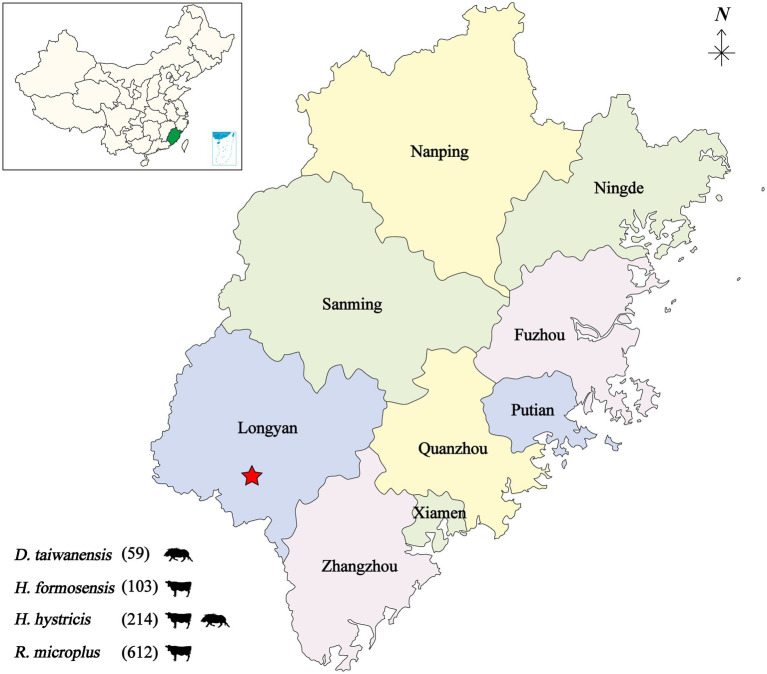
Geographic map showing the tick sampling information. The tick collection site is indicated by a red star, annotated with tick species and infesting animals. *D, Dermacentor*; *H, Haemaphysalis*; *R, Rhipicephalus*.

From 2019 to 2020, a total of 988 bloodsucking ticks were collected from cattle and wild boar in seven villages of the Meihua Mountain Nature Reserve, Fujian province, Southeastern China. The tick species were determined as *Dermacentor taiwanensis* (59, 8 pools), *Haemaphysalis hystricis* (214, 18 pools), *Haemaphysalis formosensis* (103, 8 pools), and *Rhipicephalus microplus* (612, 100 pools). The 988 ticks were divided into 134 individual pools based on tick species and host animals (Figure 1).

**Figure 2:**
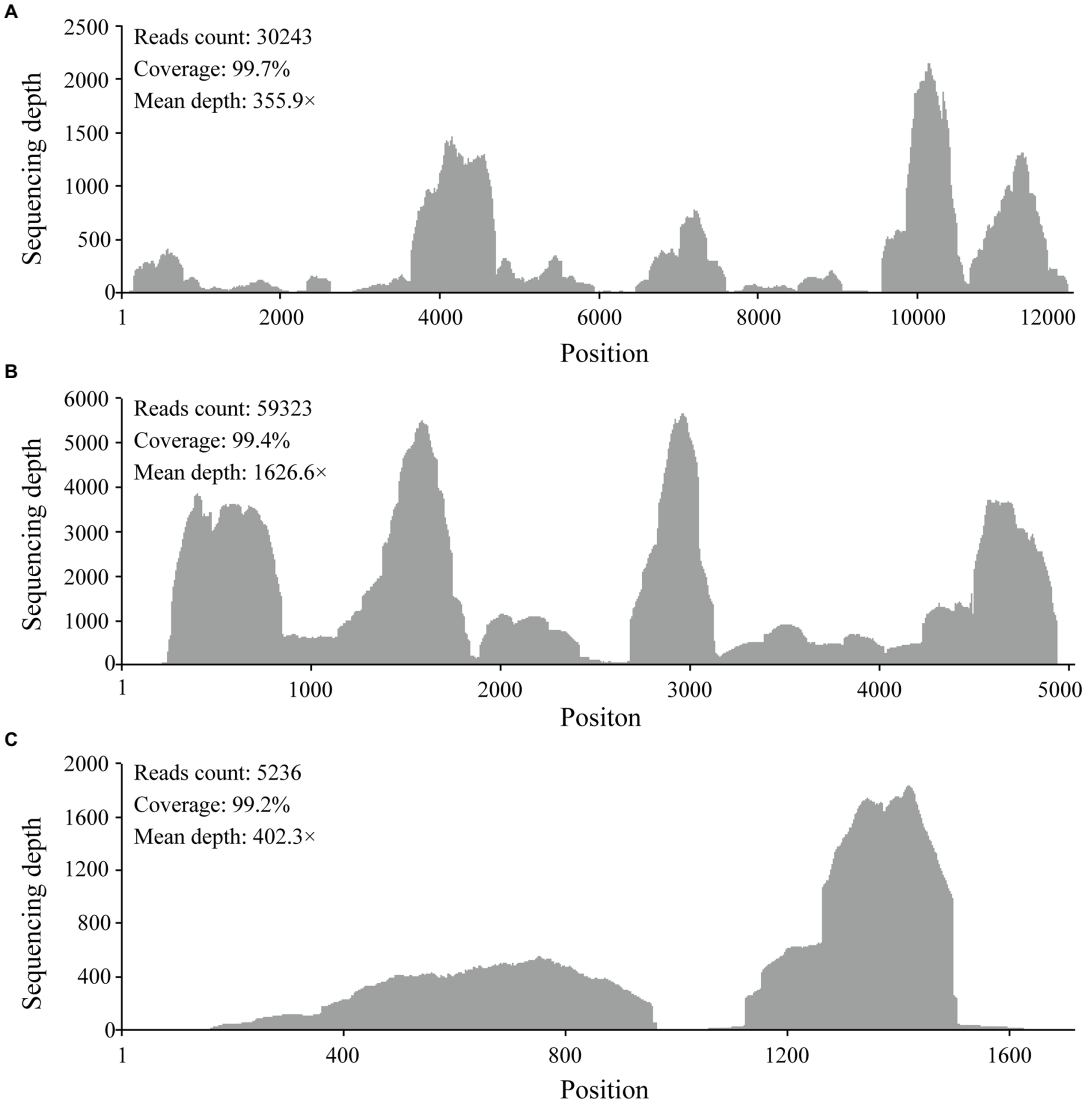
Mapped reads count plot of the MHMV genome. **(A)** L segment; **(B)** M segment; **(C)** S segment. The histograms show the depth at a given base position.

RNA from 34 pools was selected based on tick species and host animals, mixed, and subjected to meta-transcriptomic sequencing. A total of 41,271,206 paired-end clean reads were generated. After removing the ribosomal and tick host reads, 14,569,764 reads were obtained and assembled. The resulting contigs were blasted against virus nucleotides and proteins, which identified 706,102 viral reads, accounting for 1.71% of total clean reads. Among the assembled contigs, 59 viral contigs were annotated as the Large (L) (32), Middle (M) (21), and Small (S) (6) segments of orthonairoviruses within the family *Nairoviridae*. The novel virus was tentatively designated as MHMV. The full-length genome was determined by RT-PCR to fill the gaps between assembled contigs and RACE to obtain the genome termini. Thereafter, a total of 94,802 reads were re-mapped to the determined MHMV genome, revealing an overall >99% genome coverage with a mean depth of >350 × (Figure 2). MHMV has a tripartite negative-sense RNA genome that comprises three segments, including an L segment encoding an RNA-dependent RNA polymerase (RdRp), an M segment encoding a GPC, and an S segment encoding a nucleocapsid protein. The nucleotide sequences of L, M, and S segments of MHMV have been deposited in GenBank under accession numbers ON184084−ON184086, respectively.

### Phylogenetic analysis of MHMV

**Figure 3:**
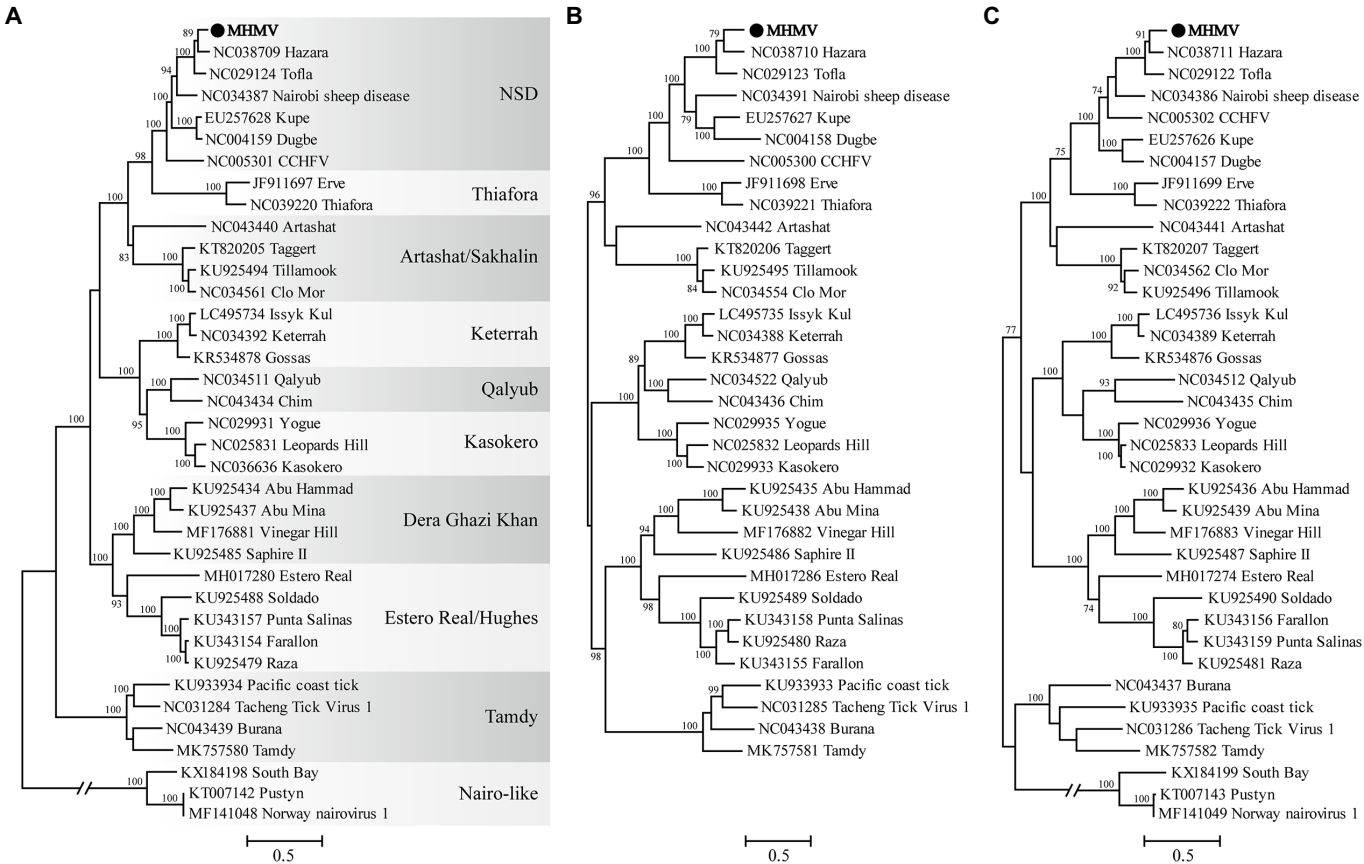
ML phylogenetic trees based on the amino acid sequences deduced from L, M, and S segments of Meihua Mountain Virus (Black dots) and other representative orthonairoviruses. **(A)** Tree inferred from the RdRp amino acid sequences. **(B)** Tree inferred from the GPC amino acid sequences. **(C)** Tree inferred from the N protein amino acid sequences. Genogroup assignments are indicated with differential shading patterns. Bootstrap support values ≥70% are indicated. Sequences are identified by their GenBank accession numbers, followed by the virus name. GenBank accession numbers of sequences used in the alignments are listed in Supplementary Table S2.

The virus sequence was preliminarily compared against Viruses reference sequences with blastn suite. The nucleotide sequences of L segment, M segment, and S segment of MHMV have the highest sequence similarity with HAZV with identities of 71.9%, 64.4%, and 69.4%, respectively (Table 1). These results support that MHMV is a novel species in genus *Orthonairovirus* according to the criteria suggested (He et al., 2022). ML phylogenetic trees reconstructed based on the amino acid sequences from L, M, and S segments of orthonairoviruses confirmed that members of the genus *Orthonairovirus* have been assigned into nine genogroups (Figure 3; Walker et al., 2016). The RdRp, GPC, and N protein trees all showed that MHMV was closely clustered with HAZV and a recently identified Orthonairovirus, TFLV (Figure 3). These three viruses formed a well-supported (100% bootstrap value at the node) small clade in the NSD genogroup, indicating MHMV is a novel member of the NSD genogroup.

**Table 1:** Amino acid sequence and nucleotide identity of MHMV RdRp (L segment), GPC (M segment), and N protein (S segment) with the NSD genogroup orthonairoviruses.

	Amino acid sequence identity (%)			Nucleotide sequence identity (%)		
	RdRp	GPC	N	L	M	S
HAZV	82.4	66.1	77.3	71.9	64.4	69.4
TFLV	83.5	64.5	75.3	71.1	63.2	69.4
NSDV	68.9	45.4	61.1	65.1	51.2	61.8
KUPV	65.6	43.2	54.7	62.5	50.3	58.5
DUGV	65.9	38.9	53.4	62.6	48.0	56.0
CCHFV	62.3	37.1	59.1	61.5	47.3	58.0

### Genome characteristics of the novel MHMV

The MHMV has a tripartite negative-sense RNA genome that comprises three segments as other orthonairoviruses. The L segment is 11,897 nt in length encoding an RdRp (3,923 aa). The M segment (4,853 nt) encodes a GPC (1,482 aa), and an S segment (1,682 nt) encodes an N protein (485 aa). The RdRp ORF is flanked by an 85 bp 5′-non-coding region (NCR) and a 40 bp 3′-NCR in the L segment. The M segment is flanked by a 337 bp 5′-NCR and a 67 bp 3′-NCR, while the S segment has a 143 bp 5′-NCR and an 81 bp 3′-NCR. All three segments shared the conserved terminal sequences with 3′-AGAGUUUCU and a reverse complementary 5′-AGAAACUCU, the typical feature of the genus *Orthonairovirus* (Kuhn et al., 2016).

The RdRp of nairoviruses contains several presumed regions and domains, including ovarian tumor-like (OTU-like) protease domain, polymerase module (pre-Motif A, motif A-E), and the Region I, Region II (a recently identified N-terminal localized Influenza-Like endonuclease domain which had a cap-snatching activity) and Region IV (Honig et al., 2004; Kinsella et al., 2004; Reguera et al., 2010). Amino acid sequence alignment of these regions confirmed that the RdRp of MHMV contains all the aforementioned highly conserved regions as other NSD genogroup members (Supplementary Figure S1).

**Figure 4:**
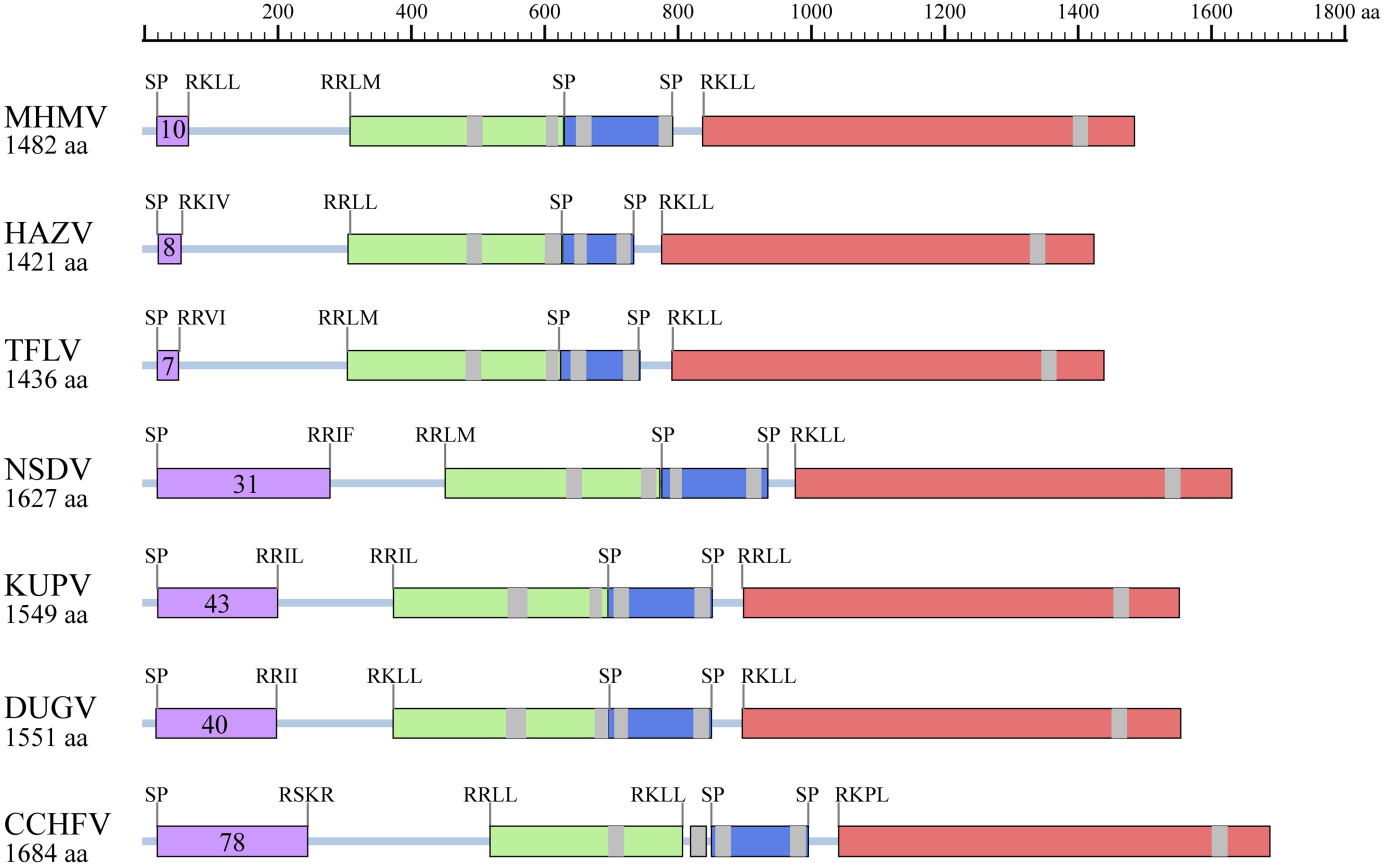
Illustration of GPC structures of the NSD genogroup viruses. Mucin-like domain (purple), Gn (light green), NSm (dark blue), and Gc (pink) are shaded correspondingly. Predicted signal peptidase (SP) cleavage sites and putative furin-like and subtilisin/kexin-isozyme-1 (SKI-I) cleavage sites are labeled. The number of predicted *O*-linked glycosylation sites in the mucin-like domain is also shown in the purple shaded box. The transmembrane domains are shaded with grey. The protein length is scaled. HAZV, Hazara virus; TFLV, Tofla virus; NSDV, Nairobi sheep disease virus; KUPV, Kupe virus; DUGV, Dugbe virus; CCHFV, Crimean-Congo hemorrhagic fever virus.

Moreover, protein domain and post-translational modification analysis of MHMV GPC showed typical organizational characteristics in terms of three predicted signal peptidase cleavage sites, three subtilisin/kexin-isozyme-1 (SKI-1) protease cleavage sites (RRKL^51^, RRLM^310^, and RKLL^837^), 5 transmembrane domains, presumable mucin-like domain located at the N-terminus, glycoprotein (Gn), a non-structural protein (NSm), and glycoprotein (Gc; Figure 4). Notably, MHMV has similar Gn and Gc structure and protein size, although a relatively lower amino acid identity with other NSD genogroup viruses (Figure 4; Table 1). Interestingly, MHMV, HAZV, and TFLV contain much shorter mucin-like domains and fewer *O*-glycosylation sites in comparison with NSDV, KUPV, DUGV, and CCHFV (Figure 4). The Gn protein of MHMV contains two *N*-glycosylation sites, two transmembrane domains, and a pair of conserved zinc-finger domains that have also been found in CCHFV (Supplementary Figure S2; Estrada and De Guzman, 2011). Similarly, three conserved *N*-glycosylation sites have been found in Gc of MHMV and other NSD group viruses (Supplementary Figure S3). A C-terminus localized transmembrane domain and two conserved *N*-glycosylation sites were also predicted in other NSD genogroup members (Supplementary Figure S3). Moreover, MHMV possesses conserved fusion loop regions as other Orthonairovirus (Supplementary Figure S3; Li et al., 2022).

Structural biology studies of N protein of nairoviruses identified two major domains, a globular head and an extended stalk, with RNA/DNA binding-associated sites. The head domains of the MHMV and other NSD genogroup viruses showed high conservation while apparent high flexibility can be seen in the stalk domain as previously reported (Supplementary Figure S4; Wang et al., 2015). In addition, the DEVD caspase-3 cleavage site motif that has been identified in CCHFV is not present in MHMV N protein (Wang et al., 2012). These results showed that MHMV shares conserved genome organization and protein structures with other NSD genogroup orthonairoviruses.

### Sequence comparison of MHMV with other NSD genogroup members

The NSD genogroup now consists of six representative species, including HAZV, TFLV, NSDV, KUPV, DUGV, and CCHFV. The pairwise alignment showed that the RdRp, GPC, and N protein of MHMV share 62.3%–83.5%, 37.1%–66.1%, and 53.4%–77.3% amino acid identity with other NSD genogroup viruses, respectively (Table 1). The RdRp amino acid sequence of MHMV has the highest identity (83.5%) with TFLV, while GPC and N both showed the highest identity to HAZV with the identity of 66.1 and 77.3%, respectively (Table 1).

Notably, the Gn protein of the NSD genogroup viruses has a similar percent identity (38.4%–66.5%) with GPC polyprotein (35.8%–66.1%; Table 2). However, the Gc part showed a significantly higher percent identity range from 44.8% to 78.0% within the genogroup (Table 2). To date, an objective species demarcation criterion of *Orthonairovirus* stays unestablished. The species demarcation criteria for *Nairoviridae* are recently proposed to be <93% identity in L protein, considering that available CCHFV strains possess a maximum amino acid distance of 6.8% (Walker et al., 2016). Thus, we tentatively designated the newly identified virus, MHMV, as a novel species of the NSD genogroup in the genus *Orthonairovirus*.

**Table 2:** Pairwise comparisons of GPC, Gn, and Gc amino acid sequences among the NSD genogroup orthonairoviruses.

	**Amino acid sequence identity (%)**					
	MHMV	HAZV	TFLV	NSDV	KUPV	DUGV
GPC						
MHMV	100					
HAZV	66.1	100				
TFLV	64.5	62.0	100			
NSDV	45.4	48.6	46.3	100		
KUPV	43.2	46.0	43.8	49.6	100	
DUGV	38.4	42.1	40.0	45.5	56.3	100
CCHFV	37.1	38.7	38.9	40.5	40.3	35.8
Gn						
MHMV	100					
HAZV	66.5	100				
TFLV	66.1	64.3	100			
NSDV	46.4	45.5	45.8	100		
KUPV	45.8	45.8	47.3	53.9	100	
DUGV	42.1	44.0	43.4	51.6	60.4	100
CCHFV	38.4	40.9	41.5	43.7	44.6	41.0
Gc						
MHMV	100					
HAZV	78.0	100				
TFLV	75.5	74.6	100			
NSDV	59.7	62.3	58.1	100		
KUPV	56.5	58.1	55.6	61.9	100	
DUGV	57.1	59.4	56.7	63.1	72.2	100
CCHFV	48.8	49.1	49.1	52.0	51.3	52.0

### Prevalence of MHMV in ticks

To estimate the prevalence of MHMV in ticks, the collected 134 individual tick pools were subjected to molecular screening of MHMV. The amplicon of RT-PCR was sequenced and two variant sequences have been deposited to Genbank under accession numbers of OP056094 and OP056095, respectively. The positive rate was 7.38% (95% CI: 3.84%–12.59%, 18 pools) in *H. hystricis*, 6.02% (95% CI: 1.85%–14.22%, eight pools) in *H. formosensis*, and 25.03% (95% CI: 9.23%–54.59%, eight pools) in *D. taiwanensis*, while only one pool of *R. microplus* was tested positive out of 100 (Table 3), suggesting that the hard ticks, *Haemaphysalis* spp. and *Dermacentor* spp., might be the predominant vectors of MHMV. However, isolation of MHMV with commonly used cell lines for NSD genogroup virus isolation was not successful.

**Table 3:** Prevalence of MHMV in tick pools screened by RT-PCR.

	No. of MHMV positive/tested pools (positive rate, 95% CI)			
	*H. hystricis*	*D. taiwanensis*	*H. formosensis*	*R. microplus*
Wild boar	8/12 (8.89, 4.04%–16.63%)	6/- (25.03, 9.23%–54.59%)	—	—
Cattle	3/6 (5.16, 1.3%–13.06%)	—	4/8 (6.02, 1.85%–14.22%)	1/100 (0.16, 0.01%–0.72%)

*D, Dermacentor; H, Haemaphysalis; R, Rhipicephalus.*

## Discussion

Tick-transmitted viral pathogens can cause deadly illnesses in animals and humans, which has posed a significant threat to public health (Madison-Antenucci et al., 2020). With the expansion of tick habitats, it is necessary to survey the pathogenic agents they carried. In this study, we took unbiased high throughput meta-transcriptomic sequencing to profile viruses carried by ticks collected from Fujian, a coastal mountainous province, Southeastern China. A novel Orthonairovirus, MHMV, was identified in bloodsucking ticks collected from cattle and wild boar. Since the viral structural proteins possess relatively low identity (<83.5% in L, <66.1% in GPC, and <77.3% in N) to its closest Orthonairovirus relatives, we herein suggest MHMV as a novel species of genus *Orthonairovirus*. Phylogenetic analysis suggested that MHMV is closely related to HAZV and belongs to the NSD genogroup in the genus *Orthonairovirus*. It shares typical genomic characteristics with other orthonairoviruses. Molecular surveillance showed that MHMV is mainly carried by the ticks of *Haemaphysalis* spp. and *Dermacentor* spp.

The expanding genus *Orthonairovirus* is now a large group with 15 highly divergent species and several unclassified nairoviruses (Garrison et al., 2020). Among them, CCHFV, the most pathogenic one, has been found in China by molecular and serological detection in ticks and humans, respectively (Sun et al., 2009; Moming et al., 2018). Recently, some novel Orthonairovirus, such as Songling virus (Ma et al., 2021), Tacheng tick virus 1(Liu et al., 2019), and Tamdy virus (strain XJ01/TAMV/China/2018; Zhou et al., 2019) have been identified in ticks collected in Northeastern and Northwestern China, respectively. The identification of MHMV in Fujian province, Southeastern China, suggested the genetic diversity and wide geographic distribution of orthonairoviruses in China. More importantly, songling virus and Tacheng tick virus 1 have been confirmed associated with human febrile illness (Liu et al., 2019; Ma et al., 2021), indicating the public health significance of Orthonairovirus. Although the pathogenicity of MHMV needs further investigation, the close phylogenetic relationship with other pathogenic Orthonairovirus still indicates its pathogenic potential to human or animal.

Previously, it has been established that the classification of the genus *Orthonairovirus* was well supported by phylogenetic relationships and structural features of GPC (Walker et al., 2016). In this study, we compared the sequence conservativity and protein structure of the NSD genogroup members. Both sequence alignment and protein structure analysis supported the genogroup classification proposed by Walker et al. (2016). Notably, we showed that the Gn amino acid sequence has a similar level of identity with the whole GPC polyprotein, while the Gc part shared an apparently higher identity. If these observations infer functional consequences in terms of pathogenesis and viral biology of the NSD genogroup members remains unclear.

We have attempted to isolate MHMV with Vero E6, SW-13, and BHK-21 cell lines that have been used for isolation of NSD genogroup viruses (CCHFV, HAZV, and TFLV; Shimada et al., 2016; Fuller et al., 2020; Dai et al., 2021). However, neither CPE nor viral RNA has been detected across several passages in cell cultures. This could be explained by that MHMV is a novel virus with low identity with known NSD genogroup viruses. Tick cell line may be a better alternative for MHMV isolation in the future since it has been successfully applied in the isolation of several novel viruses from ticks (Jia et al., 2019; Kholodilov et al., 2020).

Taken together, a novel Orthonairovirus was identified in ticks collected in the Fujian province of China through unbiased RNA sequencing, which expands the knowledge about the genetic diversity of orthonairoviruses in China.

## Data availability statement

The datasets presented in this study can be found in online repositories. The names of the repository/repositories and accession number(s) can be found at: https://www.ncbi.nlm.nih.gov/genbank/, ON184084, ON184085, and ON184086.

## Author contributions

XZ and CC sampled the ticks. XZ, H-YL, and M-CP prepared the nucleic acid samples and gathered the molecular data. XZ, J-WS, and F-QH performed the bioinformatics analyses and drafted the manuscript. F-QH and M-FS conceived the study and revised the manuscript. All authors contributed to the article and approved the submitted version.

## Funding

This research was funded by the Guangdong Basic and Applied Basic Research Foundation (2020A1515011575, 2021A1515110450) and the Natural Science Foundation of Fujian Province (2021J05235).

## Conflict of interest

The authors declare that the research was conducted in the absence of any commercial or financial relationships that could be construed as a potential conflict of interest.

## Publisher’s note

All claims expressed in this article are solely those of the authors and do not necessarily represent those of their affiliated organizations, or those of the publisher, the editors and the reviewers. Any product that may be evaluated in this article, or claim that may be made by its manufacturer, is not guaranteed or endorsed by the publisher.

## Supplementary material

The Supplementary material for this article can be found online at: https://www.frontiersin.org/articles/10.3389/fmicb.2022.977405/full#supplementary-material

## Footnotes

^1^ https://epitools.ausvet.com.au/ppvariablepoolsize

^2^ https://mafft.cbrc.jp/alignment/server/

^3^ https://espript.ibcp.fr/ESPript/cgi-bin/ESPript.cgi
