# Supplementary material for: Genomic characterization and phylogenetic analysis of a novel Nairobi sheep disease genogroup Orthonairovirus from ticks, Southeastern China
Source: Front Microbiol. 2022 Aug 25;13:977405. doi: 10.3389/fmicb.2022.977405 (PMC9453679; doi:10.3389/fmicb.2022.977405)
Supplement: Supplementary file 1 [file Data_Sheet_1.pdf]

Supplementary Material

1 Supplementary Figures and Tables

1.1 Supplementary Figures

(A) OTU-like protease domain

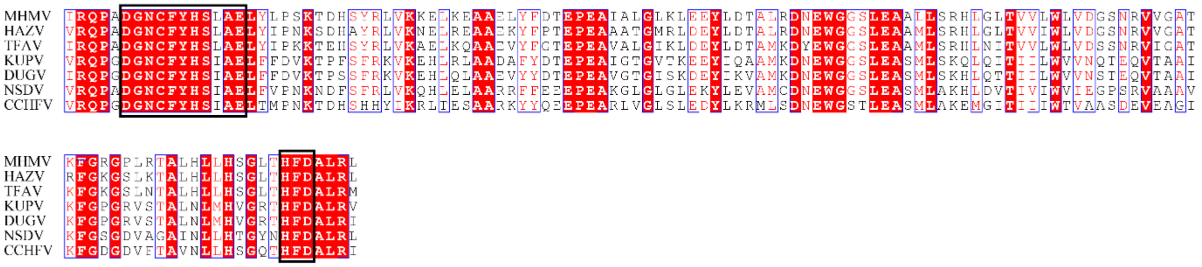

(B) Region I

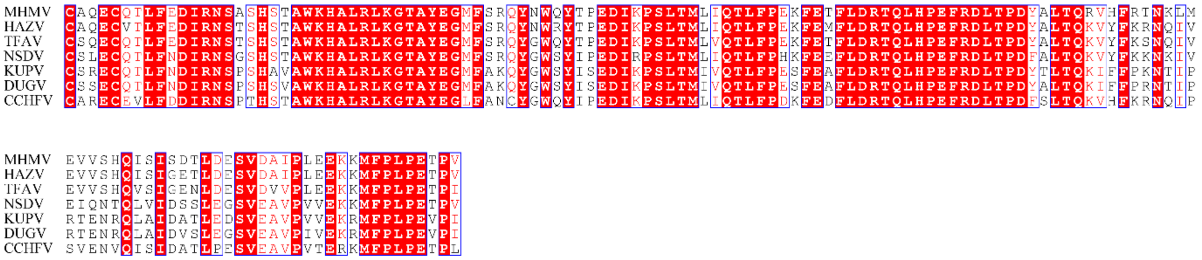

(C) Region II

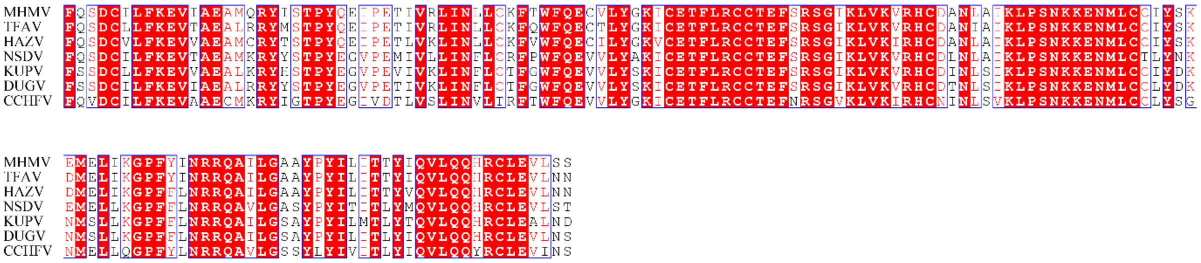

(D) Polymerase module

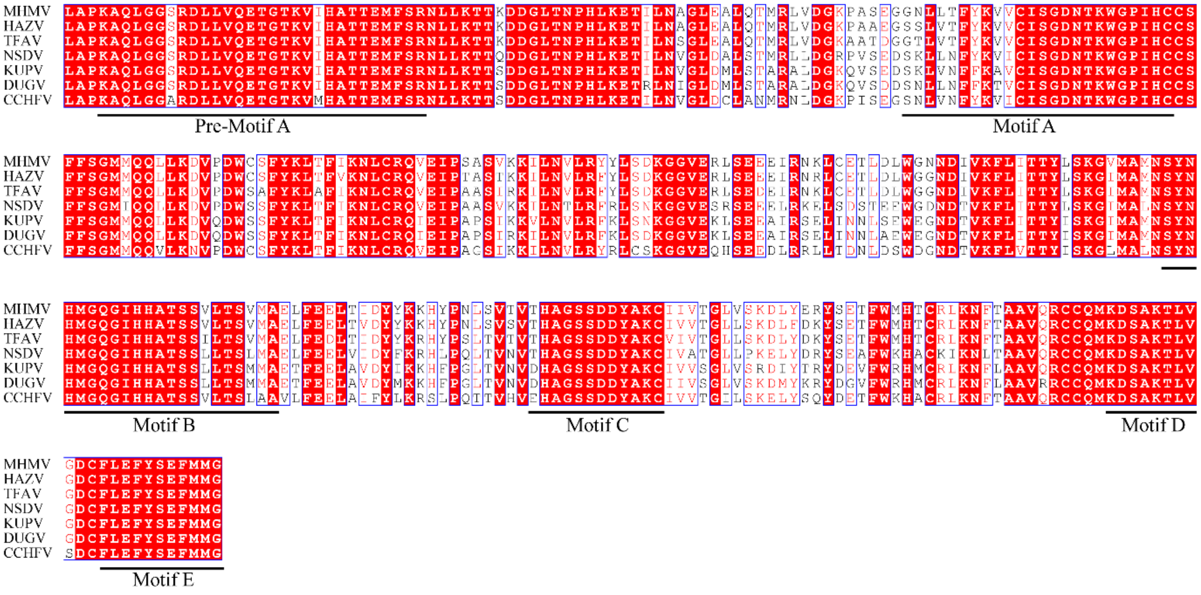

(E) Region IV

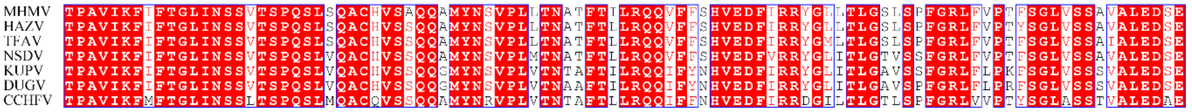

Supplementary Figure 1. Amino acid sequence alignment of the RdRp conserved regions of NSD genogroup viruses and MHMV. Conserved bases are shaded red. **(A)** Ovarian tumor domain (OTU)-like cysteine proteases motif, conserved residues are boxed; **(B)** Region I; **(C)** Region II; **(D)** Polymerase module, pre-Motif A and Motif A–E are underlined; **(E)** Region IV are highly conserved in bunya- and arenaviruses. HAZV, Hazara virus; TFLV, Tofla virus; NSDV, Nairobi sheep disease virus; KUPV, Kupe virus; DUGV, Dugbe virus; CCHFV, Crimean-Congo hemorrhagic fever virus.

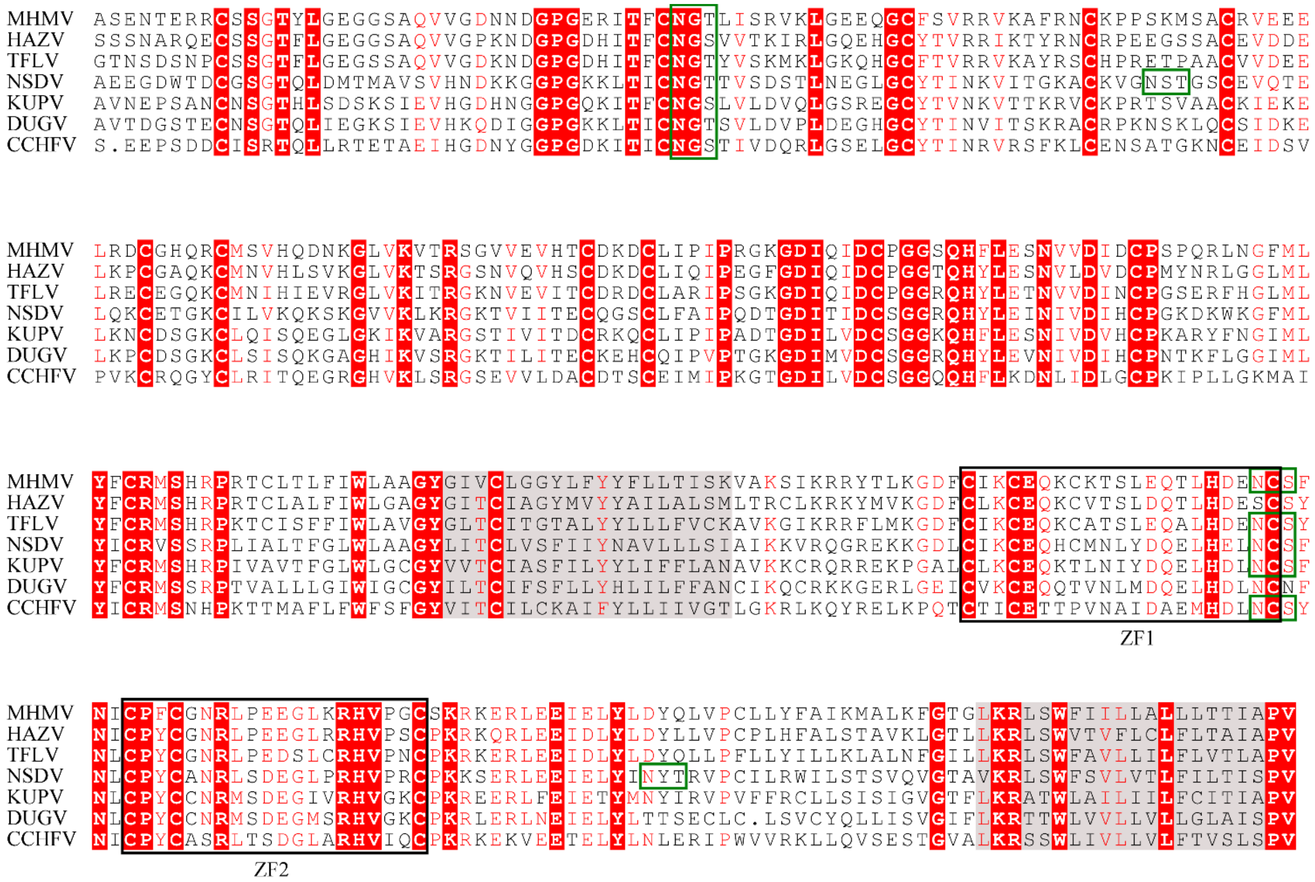

Supplementary Figure 2. Amino acid sequence alignment of the Gns of NSD genogroup viruses and MHMV. Conserved bases are shaded red. Two transmembrane domains are shaded gray and two C-terminus localized zinc-finger motifs are boxed with black line. Putative N-glycosylation sites are boxed with green. HAZV, Hazara virus; TFLV, Tofla virus; NSDV, Nairobi sheep disease virus; KUPV, Kupe virus; DUGV, Dugbe virus; CCHFV, Crimean-Congo hemorrhagic fever virus.

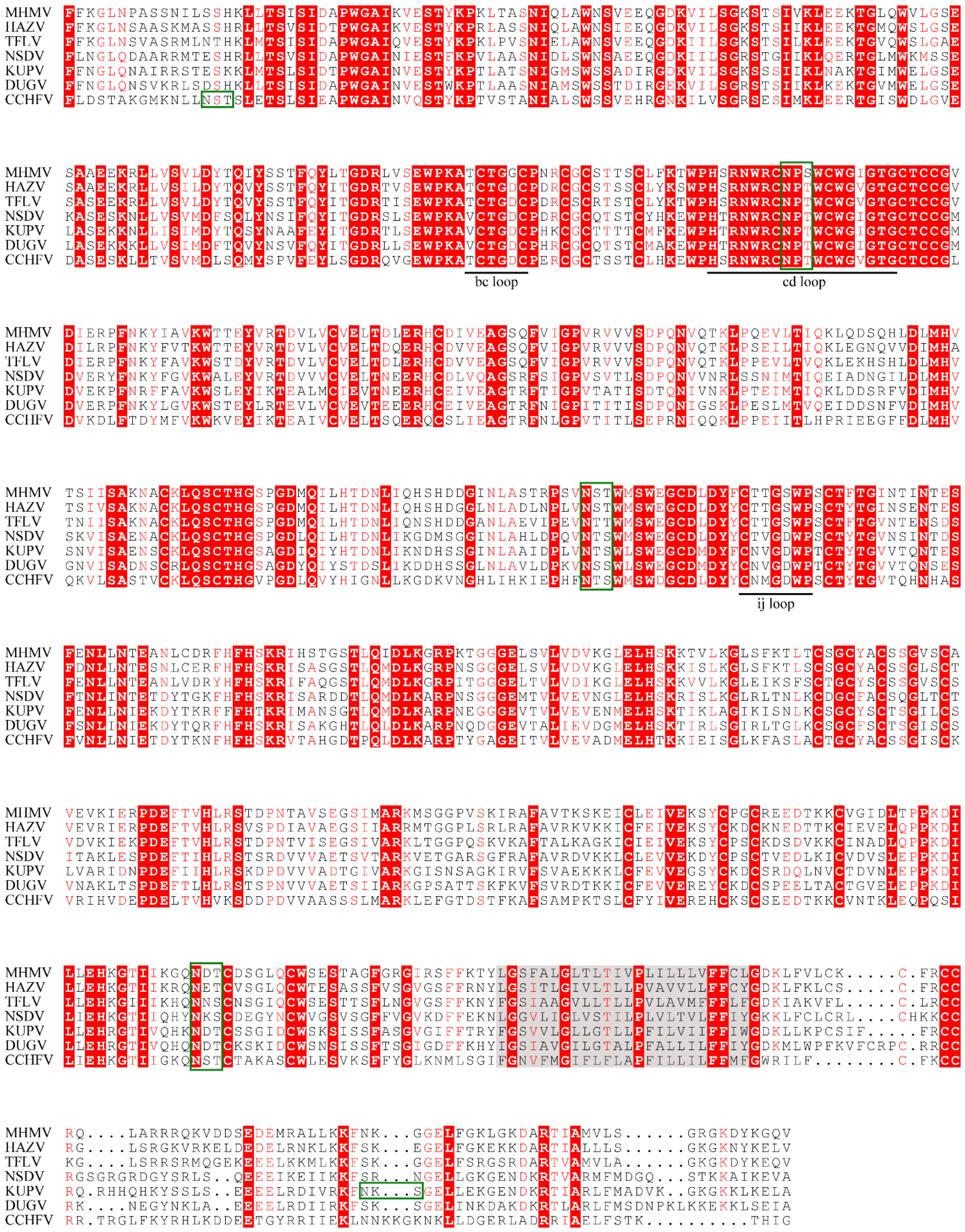

Supplementary Figure 3. Amino acid sequence alignment of the Gcs of NSD genogroup viruses and MHMV. Conserved bases are shaded red. Transmembrane domain is shaded gray and putative

N-glycosylation sites are boxed with green. Fusion loops are underlined. HAZV, Hazara virus; TFLV, Tofla virus; NSDV, Nairobi sheep disease virus; KUPV, Kupe virus; DUGV, Dugbe virus; CCHFV, Crimean-Congo hemorrhagic fever virus.

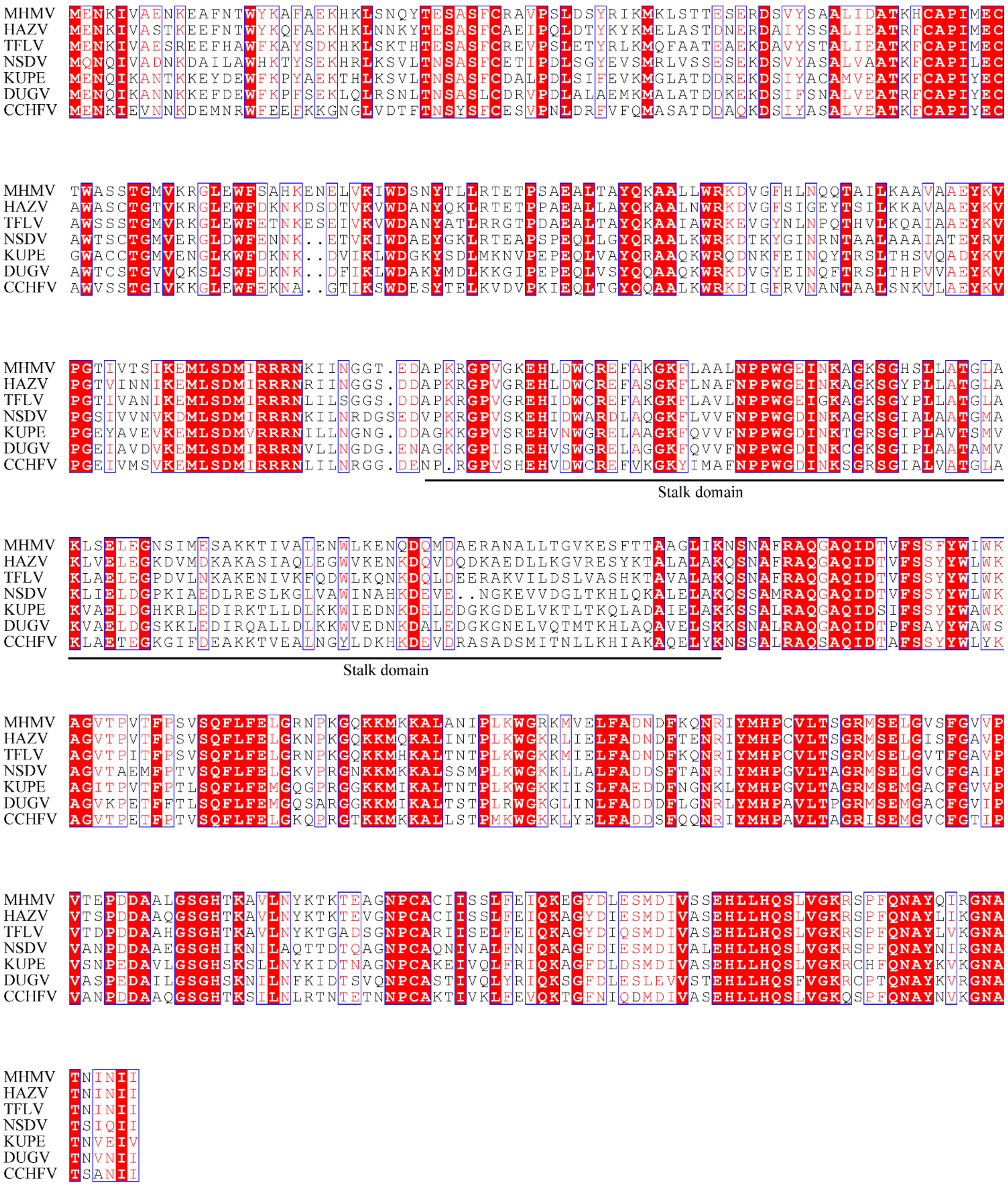

Supplementary Figure 4. Amino acid sequence alignment of the nucleoproteins of NSD genogroup viruses and MHMV. Conserved bases are shaded red. The flexible stalk domain is underlined. HAZV, Hazara virus; TFLV, Tofla virus; NSDV, Nairobi sheep disease virus; KUPV, Kupe virus; DUGV, Dugbe virus; CCHFV, Crimean-Congo hemorrhagic fever virus.

1.2 Supplementary Tables

Supplementary Table 2 Primers for tick identification and virus prevalence screening in this study.

Primer Name	Sequence	Application
Tick-F	5'-CTGCTCAATGATTTTTTAAATTGCTGTGG-3'	Tick species identification
Tick-R	5'-CCGGTCTGAACTCAGATCAAGT-3'	Tick species identification
L-F1	5'-TGGCTTGATGTCTTCGGGTG-3'	Molecular screening of MHMV
L-R1	5'-GCAGCATTGGGAACGTTCTTT-3'	Molecular screening of MHMV
L-F2	5'-TGCCTTCGTAAGCAGTTCCC-3'	Molecular screening of MHMV
L-R2	5'-ACGTTCTTTCTTACGCACACA-3'	Molecular screening of MHMV

Supplementary Table 2 Tick pools subjected to meta-transcriptomic sequencing in this study.

Pool number	Host animal	Number of ticks	Tick species
1	wild boar	1	*Dermacentor taiwanensis*
2	wild boar	10	*Dermacentor taiwanensis*
3	wild boar	8	*Dermacentor taiwanensis*
4	wild boar	5	*Dermacentor taiwanensis*
5	wild boar	1	*Dermacentor taiwanensis*
6	wild boar	9	*Dermacentor taiwanensis*
7	wild boar	10	*Dermacentor taiwanensis*
8	wild boar	15	*Dermacentor taiwanensis*
9	cattle	15	*Haemaphysalis formosensis*
10	cattle	15	*Haemaphysalis formosensis*
11	cattle	15	*Haemaphysalis formosensis*
12	cattle	10	*Haemaphysalis formosensis*
13	cattle	11	*Haemaphysalis formosensis*
14	cattle	20	*Haemaphysalis formosensis*
15	cattle	16	*Haemaphysalis formosensis*
16	cattle	1	*Haemaphysalis formosensis*
17	wild boar	10	*Haemaphysalis hystricis*
18	wild boar	6	*Haemaphysalis hystricis*
19	wild boar	12	*Haemaphysalis hystricis*
20	cattle	15	*Haemaphysalis hystricis*
21	cattle	8	*Haemaphysalis hystricis*
22	cattle	16	*Haemaphysalis hystricis*
23	cattle	5	*Haemaphysalis hystricis*
24	cattle	13	*Haemaphysalis hystricis*
25	cattle	15	*Haemaphysalis hystricis*
26	wild boar	20	*Haemaphysalis hystricis*

27	wild boar	12	*Haemaphysalis hystricis*
28	wild boar	13	*Haemaphysalis hystricis*
29	wild boar	10	*Haemaphysalis hystricis*
30	wild boar	4	*Haemaphysalis hystricis*
31	wild boar	15	*Haemaphysalis hystricis*
32	wild boar	16	*Haemaphysalis hystricis*
33	wild boar	12	*Haemaphysalis hystricis*
34	wild boar	12	*Haemaphysalis hystricis*

Supplementary Table 3. Viruses and GenBank accession numbers of sequences used in phylogenetic and other comparative analyses.

Virus (strain)	Segment	Genbank accession
Abu Hammad virus (Art 1194)	L	KU925434.1
Abu Hammad virus (Art 1194)	M	KU925435.1
Abu Hammad virus (Art 1194)	S	KU925436.1
Abu Mina virus (EG AN 4996)	L	KU925437.1
Abu Mina virus (EG AN 4996)	M	KU925438.1
Abu Mina virus (EG AN 4996)	S	KU925439.1
Artashat orthonairovirus	L	NC_043440.1
Artashat orthonairovirus	M	NC_043442.1
Artashat orthonairovirus	S	NC_043441.1
Burana virus	L	NC_043439.1
Burana virus	M	NC_043438.1
Burana virus	S	NC_043437.1
Chim orthonairovirus	L	NC_043434.1
Chim orthonairovirus	M	NC_043436.1
Chim orthonairovirus	S	NC_043435.1
Clo Mor virus (ScotAr7)	L	NC_034561.1
Clo Mor virus (ScotAr7)	M	NC_034554.1
Clo Mor virus (ScotAr7)	S	NC_034562.1
Crimean-Congo hemorrhagic fever virus	L	NC_005301.2
Crimean-Congo hemorrhagic fever virus	M	NC_005300.2
Crimean-Congo hemorrhagic fever virus	S	NC_005302.2
Dugbe virus (ArD 44313)	L	NC_004159.1
Dugbe virus (ArD 44313)	M	NC_004158.1
Dugbe virus (ArD 44313)	S	NC_004157.1
Erve virus	L	JF911697.1
Erve virus	M	JF911698.1
Erve virus	S	JF911699.1
Estero Real orthobunyavirus (K329)	L	MH017280.1

Estero Real orthobunyavirus (K329)	M	MH017286.1
Estero Real orthobunyavirus (K329)	S	MH017274.1
Farallon virus (CalAr846)	L	KU343154.1
Farallon virus (CalAr846)	M	KU343155.1
Farallon virus (CalAr846)	S	KU343156.1
Gossas virus (DakAnD 401)	L	KR534878.1
Gossas virus (DakAnD 401)	M	KR534877.1
Gossas virus (DakAnD 401)	S	KR534876.1
Hazara virus (JC280)	L	NC_038709.1
Hazara virus (JC280)	M	NC_038710.1
Hazara virus (JC280)	S	NC_038711.1
Issyk-Kul virus (LEIV-315K)	L	LC495734.1
Issyk-Kul virus (LEIV-315K)	M	LC495735.1
Issyk-Kul virus (LEIV-315K)	S	LC495736.1
Kasokero virus (Z-52963)	L	NC_029932.1
Kasokero virus (Z-52963)	M	NC_029933.1
Kasokero virus (Z-52963)	S	NC_036636.1
Keterrah virus (P61361)	L	NC_034392.1
Keterrah virus (P61361)	M	NC_034388.1
Keterrah virus (P61361)	S	NC_034389.1
Kupe virus (K611)	L	EU257628.1
Kupe virus (K611)	M	EU257627.1
Kupe virus (K611)	S	EU257626.1
Leopards Hill (11SB17)	L	NC_025831.1
Leopards Hill (11SB17)	M	NC_025832.1
Leopards Hill (11SB17)	S	NC_025833.1
Nairobi sheep disease virus (Jilin)	L	NC_034387.1
Nairobi sheep disease virus (Jilin)	M	NC_034391.1
Nairobi sheep disease virus (Jilin)	S	NC_034386.1
Norway nairovirus 1 (NOR/B1V/Tofte/2014)	L	MF141049.1
Norway nairovirus 1 (NOR/B1V/Tofte/2014)	S	MF141048.1
Pacific coast tick nairovirus (Docc2011cons)	L	KU933934.1
Pacific coast tick nairovirus (Docc2011cons)	M	KU933933.1
Pacific coast tick nairovirus (Docc2011cons)	S	KU933935.1
Punta Salinas virus (CalAr888)	L	KU343157.1
Punta Salinas virus (CalAr888)	M	KU343158.1
Punta Salinas virus (CalAr888)	S	KU343159.1
Pustyn virus (IxRic1)	L	KT007142.1
Pustyn virus (IxRic1)	S	KT007143.1
Qalyub virus (ErAg370)	L	NC_034511.1
Qalyub virus (ErAg370)	M	NC_034522.1

Qalyub virus (ErAg370)0	S	NC_034512.1
Raza virus (829)	L	KU925479.1
Raza virus (829)	M	KU925480.1
Raza virus (829)	S	KU925481.1
Saphire II virus (RML 52323-14)	L	KU925485.1
Saphire II virus (RML 52323-14)	M	KU925486.1
Saphire II virus (RML 52323-14)	S	KU925487.1
Soldado virus (TRVL 52214)	L	KU925488.1
Soldado virus (TRVL 52214)	M	KU925489.1
Soldado virus (TRVL 52214)	S	KU925490.1
South Bay virus (SC1)	L	KX184198.1
South Bay virus (SC1)	S	KX184199.1
Tacheng Tick Virus 1	L	NC_031284.1
Tacheng Tick Virus 1	M	NC_031285.1
Tacheng Tick Virus 1	S	NC_031286.1
Taggert virus	L	KT820205.1
Taggert virus	M	KT820206.1
Taggert virus	S	KT820207.1
Tamdy virus (XJ01)	L	MK757580.1
Tamdy virus (XJ01)	M	MK757581.1
Tamdy virus (XJ01)	S	MK757582.1
Thiafora virus (AnD 11411)	L	NC_039220.1
Thiafora virus (AnD 11411)	M	NC_039221.1
Thiafora virus (AnD 11411)	S	NC_039222.1
Tillamook virus (RML 86)	L	KU925494.1
Tillamook virus (RML 86)	M	KU925495.1
Tillamook virus (RML 86)	S	KU925496.1
Tofla virus	L	NC_029124.1
Tofla virus	M	NC_029123.1
Tofla virus	S	NC_029122.1
Vinegar Hill virus (CS1499)	L	MF176881.1
Vinegar Hill virus (CS1499)	M	MF176882.1
Vinegar Hill virus (CS1499)	S	MF176883.1
Yogue virus (DakAnD 56)	L	NC_029931.1
Yogue virus (DakAnD 56)	M	NC_029935.1
Yogue virus (DakAnD 56)	S	NC_029936.1
